# IMPACT OF OBJECTIVE AND SUBJECTIVE SEP ON AGING TRAJECTORIES OF FUNCTIONAL CAPACITY

**DOI:** 10.1093/geroni/igac059.876

**Published:** 2022-12-20

**Authors:** Deborah Finkel, Charlotta Nilsen, Shireen Sindi, Ingemar Kåreholt

**Affiliations:** Indiana University Southeast, New Albany, Indiana, United States; Jönköping University, Jönköping, Jonkopings Lan, Sweden; Karolinska Institutet, Solna, Stockholms Lan, Sweden; Jönköping University, Jönköping, Jonkopings Lan, Sweden

## Abstract

Long-term stress is associated with adverse health outcomes in aging. It is important to identify not only factors that influence functioning in late adulthood, such as accumulated stress, but also the timing of such factors. The aim of the current analysis was to examine how socioeconomic stressors throughout the life course are associated with aging in functional capacity. Data were available from 740 adults ranging in age from 40 to 83 at intake (mean = 62.4, SD = 8.2) who participated in up to 8 waves of data collection (mean = 3.9, SD = 2.4). A Functional Aging Index (FAI) was created by combining measures of sensory, pulmonary, gait, and grip functioning. Both childhood and adulthood measures of objective socioeconomic position (SEP) and perceived SEP (financial strain) were available. Latent growth curve models (corrected for twinness) were used to estimate the trajectory of change in FAI over age and the impact of child and adult SEP measures on the trajectories. Results indicated that both childhood and adult objective SEP independently influenced rates of change in FAI in adulthood: higher SEP was associated with higher mean functioning and slower rates of decline. In combination, model fitting indicated that if SEP is above the median in adulthood, then childhood SEP has no impact on FAI trajectories; however, if SEP is below the median in adulthood, then childhood SEP can play a role. In addition, results indicated possible long-term effects of childhood financial strain on rates of change in FAI in adulthood.

